# A five-day inpatient EMDR treatment programme for PTSD: pilot study

**DOI:** 10.1080/20008198.2018.1425575

**Published:** 2018-02-05

**Authors:** Mayaris Zepeda Méndez, Mirjam J. Nijdam, F. Jackie June ter Heide, Niels van der Aa, Miranda Olff

**Affiliations:** ^a^ Foundation Centrum ’45 | partner in Arq Psychotrauma Expert Group, Diemen, The Netherlands; ^b^ Department of Psychiatry, Academic Medical Center at the University of Amsterdam, Amsterdam, The Netherlands; ^c^ Arq Psychotrauma Expert Group, Diemen, The Netherlands

**Keywords:** Posttraumatic stress disorder, intensive treatment, trauma-focused psychotherapy, eye movement desensitization and reprocessing, feasibility, preliminary effect, yoga, 创伤后应激障碍，强化治疗，创伤中心心理治疗，眼动脱敏和再加工，可行性，早期效果，瑜伽, Trastorno de estrés postraumático, tratamiento intensivo, psicoterapia centrada en el trauma, desensibilización y reprocesamiento por movimientos oculares, viabilidad, efecto preliminar, yoga, • This pilot study investigated the potential benefits of a five-day inpatient treatment programme with EMDR and trauma-informed yoga for patients with PTSD. • After two weeks, nine out of 12 patients reported a reliable change in self-reported PTSD symptoms. • Although further research is needed to properly investigate the effects, the treatment programme was promising in showing a reduction in symptoms in a short time frame.

## Abstract

**Background**: Trauma-focused psychotherapies for posttraumatic stress disorder (PTSD) have been demonstrated to be efficacious, but also have considerable non-response and dropout rates. Intensive treatment may lead to faster symptom reduction, which may contribute to treatment motivation and thereby to reduction of dropout.

**Objective**: The aim of the current study was to investigate the feasibility and preliminary effectiveness of an intensive five-day inpatient treatment with Eye Movement Desensitization and Reprocessing (EMDR) and trauma-informed yoga for patients with PTSD.

**Method**: A non-controlled pilot study with 12 adult patients with PTSD was conducted. At baseline the PTSD diagnosis was assessed with the Clinician-Administered PTSD Scale (CAPS-5) and comorbid disorders with the Mini International Neuropsychiatric Interview (MINI). Primary outcome was self-reported PTSD symptom severity (PTSD Check List for DSM-5; PCL-5) measured at the beginning of day 1 (T1), at the end of day 5 (T2) and at follow-up on day 21 (T3). Reliable change indexes (RCI) and clinically significant changes were calculated.

**Results**: From T1 to T3, PTSD symptoms significantly improved with a large effect size (Cohen’s *d* = 0.91). Nine of the 11 patients who completed treatment showed reliable changes in terms of self-reported PTSD. At T3, two of the patients no longer met criteria for PTSD as measured with the PCL-5. One patient dropped out after the first day. No serious adverse events occurred.

**Conclusions**: The majority of patients in our pilot study experienced symptom reduction consistent with reliable changes in this five-day inpatient treatment with EMDR and yoga. Randomized controlled trials – with longer follow up periods – are needed to properly determine efficacy and efficiency of intensive clinical treatments for PTSD compared to regular treatment. This is one of the first studies to show that intensive EMDR treatment is feasible and is indicative of reliable improvement in PTSD symptoms in a very short time frame.

## Abstract (es)

**Planteamiento**: Se ha demostrado que las psicoterapias centradas en el trauma para el TEPT son eficaces, pero también tienen tasas considerables de falta de respuesta y abandono. El tratamiento intensivo puede llevar a una reducción más rápida de los síntomas, lo que puede contribuir a la motivación para el tratamiento y, por lo tanto, a la reducción del abandono.

**Objetivo**: El objetivo del presente estudio fue investigar la viabilidad y eficacia preliminar de un tratamiento intensivo de 5 días para pacientes hospitalizados con Desensibilización y Reprocesamiento por Movimientos Oculares (EMDR) y yoga basado en el trauma para pacientes con TEPT.

**Método**: Se realizó un estudio piloto no controlado con 12 pacientes adultos con TEPT. Al inicio del estudio, se evaluó el diagnóstico de TEPT con el CAPS-5 y los trastornos comórbidos con el MINI. El resultado principal fue la gravedad de los síntomas de PTSD por medio de autoinforme (PCL-5) medida al comienzo del día 1 (T1), al final del día 5 (T2) y en el seguimiento el día 21 (T3). Se calcularon los índices de cambio fiable (RCI, por sus siglas en inglés) y los cambios clínicamente significativos.

**Resultados**: De T1 a T3, los síntomas de TEPT mejoraron significativamente con un tamaño de efecto grande (*d* de Cohen = 0,91) y 9 de los 11 pacientes que completaron el tratamiento mostraron cambios fiables en términos de auto-informes de TEPT. En T3, dos de los pacientes ya no cumplían los criterios para el trastorno de estrés postraumático medido con el PCL-5. Un paciente se retiró después del primer día. No se produjeron eventos adversos graves.

**Conclusiones**: la mayoría de los pacientes en nuestro estudio piloto experimentaron una reducción de síntomas consistente con cambios fiables en este tratamiento de 5 días con EMDR y yoga. Se necesitan ensayos controlados aleatorios, con períodos de seguimiento más largos, para determinar adecuadamente la eficacia y la eficiencia de los tratamientos clínicos intensivos para el TEPT en comparación con el tratamiento habitual. Este es uno de los primeros estudios que demuestra que el tratamiento intensivo con EMDR es factible y es indicativo de una mejora fiable en los síntomas de TEPT después de un período de tiempo muy corto.

## Abstract (zh)

**背景：**创伤中心的 PTSD心理治疗已被证实有效，但同时也有不容忽视的无效者和脱落率。

**目标：**本研究的目的是考察用于治疗PTSD 的一个为期5天的住院病人强化治疗的可行性和早期有效性，治疗中使用眼动脱敏和再强化（EMDR）和创伤瑜伽。

**方法：**在无控制组实验中使用12名成年PTSD病人。在基线期，使用 CAPS-5进行PTSD 诊断，使用 MINI 进行工病诊断。主要结果使用自评的 PTSD 症状严重度（PCL-5），在第一天开始（T1），第5天结束（T2）和第21天的追踪期（T3）。计算可靠改变指数（RCI）和临床显著改变。

**结果：**从 T1到T3的 PTSD 症状显著改善，效应值较大(Cohen’s d = 0.91) 。11个完整完成治疗的病人中的9个显示了自评PTSD的可靠改变。在 T3，两个病人不再符合 PCL-5的PTSD 诊断标准。一个病人在第一天之后脱落。没有出现严重的负面事件。

**结论：**大部分的病人在我们的试验研究中体验了症状减轻，并在使用 EMDR和瑜伽的5天

住院病人治疗中得到保持。还需要（更长追踪期的）随机对照试验将这个 PTSD强化临床治疗和普通治疗进行对比，对其效果和效率进行适当的验证。这是展示强化 EMDR 治疗可行性的比较早的研究，在比较短的时间框架下显示了 PTSD 症状可靠的改善。

Posttraumatic stress disorder (PTSD; American Psychiatric Association, 2013) is a well-documented psychological response to life-threatening and extremely distressing events. In the Netherlands, the lifetime prevalence of any potential trauma has been found to be 80.7% and the lifetime prevalence of PTSD is 7.4% (De Vries & Olff, 2009). PTSD is associated with an array of comorbid mental and somatic health-related problems. Eighty percent of PTSD patients have been found to have one or more comorbid psychiatric disorders, such as depression, substance abuse or anxiety disorders (Brady, Killeen, Brewerton, & Lucerini, 2000). Serious health problems often co-occur with PTSD, such as chronic pain and circulatory and musculoskeletal symptoms (Pacella, Hruska, & Delahanty, 2013). Consequently, PTSD has a large impact on the social and occupational functioning of patients (Bisson et al., 2007).

Trauma-focused cognitive behavioural therapy (TF-CBT) and eye movement desensitization and reprocessing therapy (EMDR) are demonstrably the most efficacious treatment methods for PTSD to date and are recommended as treatments of choice in several guidelines (Bisson et al., 2007; National Institute of Clinical Excellence [NICE], 2015). However, several reviews and meta-analyses have shown that these treatments have large dropout and considerable non-response rates. In a review of treatment efficacy, Bradley, Greene, Russ, Dutra, and Westen (2005) report that the recovery rate of patients who entered treatment was only 56%, while 67% of treatment completers no longer met criteria for PTSD. For specific populations, such as military veterans, the recovery rate is even lower. Only 40% of military veterans lose their PTSD diagnosis after receiving trauma-focused treatment (Bradley et al., 2005; Steenkamp, Litz, Hoge, & Marmar, 2015). Imel, Laska, Jakupcak, and Simpson (2013) found an average dropout of 18% among 44 studies, yet indicated that dropout rates varied quite dramatically across studies. Schottenbauer, Glass, Arnkoff, Tendick, and Gray (2008) described a range of studies in their review, including small sample studies and non-controlled studies, and identified a broad range of dropout from 0 to 54% and non-responders ranging from 0 to 89%.

As these large rates of dropout and non-response to treatment show, it is imperative to find ways to make evidence-based treatments more effective (see Olff et al., 2015). Several studies (Haagen, Smid, Knipscheer, & Kleber, 2015; Tarrier, Sommerfield, Pilgrim, & Faragher, 2000) suggested that the most important factor associated with a negative treatment outcome is the number of attended trauma-focused therapy sessions. Inconsistency of therapy attendance can be hypothesized to be related to avoidance behaviour. One way of improving the effects of trauma-focused treatments may therefore be to offer treatment in a highly condensed, intensive form. Intensified treatment is hypothesized to lead to faster symptom reduction, which may enhance attendance and reduce dropout. Intensive forms of treatment may contribute to meeting patients’ needs and expectations (Schnyder et al., 2015). An intensive form of treatment might also be more suitable for patients who are prone to benefit less from regular trauma-focused treatments, like the veteran population. Previous unsuccessful treatment may result in demoralization and negative expectations regarding future treatments, whereas faster symptom reduction in intensive treatment may facilitate motivation for and completion of treatment.

Several case studies (Blount, Cigrang, Foa, Ford, & Peterson, 2014; Hendriks, De Kleine, Van Rees, Bult, & Van Minnen, 2010) have investigated the feasibility, tolerability and effectiveness of intensive forms of trauma-focused therapy and have shown promising results. A randomized controlled trial with a seven-day intensive cognitive therapy for PTSD by Ehlers et al. (2014) showed that this treatment programme resulted in a level of symptom reduction similar to cognitive therapy conducted over 12 weekly sessions. A comparison between intensive cognitive therapy for PTSD and regular weekly treatment in a clinical setting suggested that the intensive format may lead to increased efficacy (Murray, El-Leithy, & Billings, 2017). Specifically for veterans, Murphy et al. (2015) tested a somewhat longer intensive treatment programme of six weeks with individual TF-CBT and found promising results. One pilot study is available in which the effects of an intensive eight-day EMDR programme were investigated in seven patients (Bongaerts, Van Minnen, & de Jongh, 2017), with large effects.

The current pilot study aimed to investigate the feasibility and preliminary effectiveness of EMDR treatment offered in an intensive five-day inpatient format. We considered EMDR promising for a short-term treatment format based on studies which suggest that EMDR results in faster symptom reduction than TF-CBT (Ironson, Freund, Strauss, & Williams, 2002; Nijdam, Gersons, Reitsma, De Jongh, & Olff, 2012). Trauma-informed yoga sessions were included in the treatment format, with the aim of facilitating emotion regulation and stress regulation by means of movement, bodily awareness and breath control. Various forms of physical activity, such as yoga, can play an important part in the treatment of PTSD, as demonstrated by the results of a recent meta-analysis (Rosenbaum et al., 2015). Preparatory sessions, supportive conversations and mild behavioural activation complemented the treatment in line with good clinical practice. We hypothesized that participation in a five-day intensive inpatient treatment with EMDR and yoga would be feasible for patients and would result in a clinically significant decrease in PTSD symptom severity.

## 1. Method

### 1.1. Participants and procedure

Twelve patients participated in this pilot study. These patients were either already in outpatient treatment and asked by their therapist to participate in the study, or were asked to participate after intake. Patients were given oral and written information about the treatment programme and study procedures, after which they gave written informed consent. Inclusion criteria were a diagnosis of PTSD according to the Clinician-Administered PTSD Scale (CAPS-5) and being motivated for brief intensive inpatient treatment. Exclusion criteria were: non-Dutch or non-English speaking, acute suicidality, acute psychosis and severe substance dependency.

The pilot study was conducted at Foundation Centrum ‘45, a national centre for specialized assessment and treatment of people with complex posttraumatic psychopathology in Oegstgeest, the Netherlands. For this study, patients stayed at the clinic for five nights and five days. Prior to the intensive treatment week, patients had a maximum of three preparatory sessions in which a treatment plan was made confirming their motivation for the programme, and stating the admission date, treatment goals and adherence to rules at the clinic. Also, psycho-education was given about PTSD and the rationale behind the treatment programme. A stress signalling plan was made in which the patient’s coping mechanisms with low, middle and high stress levels were identified so that the therapists at the clinic were informed about individual coping strategies. In addition to this stress signalling plan, some simple stress regulation techniques were discussed for use at the clinic. Patients also selected the three to five traumatic memories which were most directly related to their PTSD symptoms, to be processed during the intensive week. Finally, patients were shown the clinic of Centrum ‘45. A baseline assessment took place before admission to the clinic (T0). Participants were assessed at the beginning of the first day (T1), at the end of the fifth day (T2; i.e. the last day of the treatment programme) and at day 21 (T3). To determine the feasibility of the programme, patients were asked to fill out a visual analogue scale (VAS) after every EMDR session, to monitor tolerability of the session.

### 1.2. Treatment

On the first day of the programme, patients received a short physical examination by a nurse specialist as part of standard clinical care, after which they made a case conceptualization with their EMDR therapist of the specific traumatic memories to be processed and the order in which they would be addressed. Depending on the duration of this discussion, patients started with EMDR in this first session or in the next one. Patients had their second EMDR session in the afternoon. During the next four days, patients had two 90-minute EMDR sessions a day. At the end of each day, the patients received one hour of trauma-sensitive yoga. If possible, patients were admitted in pairs and, if this was the case, they received their yoga session together. All other treatment components were offered individually. Patients were encouraged to exercise between sessions, either lightly by walking or more intensively with fitness. At the beginning and the end of the day, they had a 15-minute supportive talk with a mental health nurse. One week after treatment, patients received a follow-up 90-minute EMDR session.

The EMDR sessions were aimed at reducing emotional distress associated with the most upsetting traumatic memories, following the Dutch version of the EMDR protocol (Beer, Groote, Oppenheim, & Ten Broeke, 2015). Standard eye movements were applied for bilateral stimulation. When the Subjective Unit of Distress (SUD) did not sufficiently decrease or dissociation occurred, more distraction was added by varying in eye movements, tapping on knees of patients or patient tapping on hands of therapist, buzzers in hands of patient, throwing a ball back and forth, or different types of body movement. The protocol allowed for cognitive interweaves regarding all cognitive themes. The decision to move to the next memory was based on how much distress the previous memory still evoked and was made in agreement with the patient. A substantial reduction in SUD was required to move to the next memory. Three to six memories were processed in accordance with the treatment plan. A team of 12 licensed psychologists and psychotherapists provided the EMDR sessions, either working alone or with two therapists alternately providing the EMDR sessions over the days. All therapists were trained at level 1 of EMDR training and at least one therapist of every pair was trained at EMDR level 2.

The trauma-informed yoga was added to the programme to enhance emotional regulation skills and stress regulation by means of bodily control during the intensive treatment (Kananian, Ayoughi, Farugie, Hinton, & Stangier, 2017; Yehuda et al., 2017). The protocol was adapted from ‘Trauma-sensitive yoga’ (Emerson & Hopper, 2011) and the ‘Yoga for the mind’ programme (Mason, 2011). The trauma-informed yoga consisted of postures, breathing exercises, guided meditation on body awareness and relaxation. Key concepts of trauma-sensitive yoga are keeping the session safe and predictable by minimizing stimuli. No music is played, the instructor stays in the same place and makes predictable movements. Sessions are low-level and consist of relatively slow movements. The language used is invitational, to encourage curiosity about bodily sensations and to increase awareness over stress-induced physical sensations.

### 1.3. Measurements

#### 1.3.1. Clinical interviews

To assess the presence of a PTSD diagnosis at baseline, the Life Events Checklist-5 (LEC-5) together with the CAPS-5 were administered (Weathers, Blake, et al., 2013; Dutch version Boeschoten, Bakker, Jongedijk, & Van Minnen, 2014). The LEC-5 is a 17-item self-report measure designed to screen for exposure to potential traumatic events meeting the A-criterion of PTSD according to DSM-5. The CAPS-5 is a structured diagnostic interview. Originally developed in 1989, the CAPS has been extensively validated and is the most widely accepted criterion measure for PTSD (Weathers, Keane, & Davidson, 2001). The CAPS-5 contains 20 items measuring DSM-5 PTSD symptoms, and 10 items measuring duration of symptoms, distress or impairment, global ratings and the dissociative subtype. The CAPS-5 provides a continuous measure of the severity of overall PTSD and of the four symptom clusters (intrusions, avoidance, negative alterations in cognition/mood, arousal and reactivity) and presence/absence of PTSD diagnosis including the dissociative subtype. Interviewers rate each diagnostic criterion on a scale from 0 (*absent*) to 4 (*extreme/incapacitating*) using information on both frequency and intensity of symptoms obtained during the interview. Items with a score of ≥ 2 are counted toward diagnosis. Psychometric studies of the CAPS-5 are currently underway; initial experience suggests that it has strong interrater reliability and is generally more user-friendly and efficient than previous versions (Weathers, Marx, Friedman, & Schnurr, 2014; Weathers et al., 2017).

To assess comorbid psychiatric disorders according to DSM at baseline, the Mini International Neuropsychiatric Interview (MINI) was used. The reliability and validity of the MINI are well established (Lecrubier et al., 1997; Sheehan et al., 1997; Van Vliet & De Beurs, 2007). Since the MINI for the DSM-5 was not yet available, the MINI Dutch Version 5.0.0 developed for DSM-IV criteria was used in this study.

#### 1.3.2. Self-report questionnaires

Primary outcome measure was the PTSD Check List for DSM-5 (PCL-5; Boeschoten, Bakker, Jongedijk, & Olff,  2014; Weathers, Litz et al., 2013), which assesses self-reported PTSD symptoms. It is one of the most frequently used self-report questionnaires for PTSD. Previous versions were extensively validated (McDonald & Calhoun, 2010; Wilkins, Lang, & Norman, 2011). The PCL-5 measures the 20 DSM-5 PTSD symptoms over the last week, rated on a 5-point Likert scale, ranging from 0 (*not at all*) to 4 (*extremely*). A total severity score (range 0–80) can be obtained by summing the item scores. A provisional PTSD diagnosis can be made by counting each item rated 2 (*moderately*) or higher. A cut-off score of 33 appears to be reasonable until further psychometric work is available (Wortmann et al., 2016). First investigation reveals good psychometric properties (Bovin et al., 2016). The Brief Symptom Checklist (BSI; De Beurs & Zitman, 2006) is a 53-item self-report inventory in which participants rate the extent to which they have been bothered (0 = *not at all*, 4 = *extremely*) by various mental health symptoms in the past week. The Dutch version of the BSI has satisfactory reliability and validity (De Beurs & Zitman, 2006).

#### 1.3.3. Feasibility

Feasibility was defined as tolerability measured by a Visual Analogue scale, and as dropping out of the treatment programme due to the sessions being intolerable. After every EMDR session, patients were asked to rate the tolerability of the session. The scales ranged from 0 (*well tolerable*) to 10 (*extremely hard to tolerate*). Furthermore, we investigated symptom increase at T2 and T3. Serious adverse events and harms, as defined as the totality of possible adverse consequences of an intervention or therapy (Ioannidis et al., 2004), were also monitored, including suicidal intent, psychotic symptoms, dissociation and self-harm.

### 1.4. Statistical analyses

To interpret the effectiveness of the treatment programme with regard to PTSD symptoms, treatment outcome was categorized into recovered, improved, unchanged and worsened from T1 to T2 and from T1 to T3. This categorization of treatment outcome was based on criteria of clinically significant change and the Reliable Change Index (RCI). Because of the small sample size this method was preferred over standard quantitative methods, since problems such as a lack of statistical power and biased results due to outliers are likely to occur using standard quantitative methods in small samples. Clinically significant change was defined as a shift from a clinical level of symptoms to a subclinical level of symptoms, following the PCL-5 cut-off score. The RCI was used to establish whether the difference between two test scores obtained at two measurement occasions reflects statistically reliable change, and was calculated conform the method described by Jacobson and Truax (1991). RCI values larger than 1.96 (or smaller than −1.96) indicate that there is a statistically reliable difference between two test scores, i.e. with 95% certainty the difference between the test scores is due to actual change (improvement or deterioration) rather than measurement error. Recovery can be defined by both a clinically significant change and a statistically reliable improvement (based on RCI). Improvement and deterioration can be defined by a statistically reliable improvement or deterioration (based on RCI) but no clinically significant change. Unchanged symptom severity can be defined by the absence of a statistically reliable change (based on RCI). RCI’s of the patients who fulfilled criteria for treatment resistance or who had shown severe worsening of symptoms during previous unsuccessful attempts of trauma-focused psychotherapy were inspected separately. Treatment resistance was defined as having received at least six months of treatment with at least 12 sessions of first-line treatment (TF-CBT, Brief Eclectic Psychotherapy for PTSD [BEPP; a form of TF-CBT] or EMDR) with less than 30% symptom reduction. Preliminary effect sizes (Cohen’s *d*) were calculated using means and standard deviations of total scores of the PCL-5 at T1–T2 and T1–T3.

## 2. Results

Demographic and clinical characteristics of the 12 patients are listed in Table 1. Of the 12 patients, five had one therapist and seven had two therapists who delivered EMDR sessions. There were no differences in outcome depending on the number of therapists. In an informal evaluation, patients said that they appreciated working with two different therapists and that the therapists often complemented each other. Between T2 and T3, three of the 11 patients were followed up with one EMDR session, and seven had a reflective session. No differences in outcome were detected between the patients who had a follow-up session with EMDR and those who did not. Table 2 shows PCL-5 scores for the total group at T1, T2 and T3.

**Table 1.** Baseline demographic and clinical characteristics of intent-to-treat sample (*N* = 12).

		*N* (%)	
Male		9 (75.0)	
Civil status	Single	2 (16.7)	
	Married/partnership	3 (25.0)	
	Married/partnership with children	7 (58.3)	
Educational level^a^	Low	3 (25.0)	
	Middle	5 (41.7)	
	High	4 (33.3)	
Trauma type	Veteran	6 (50.0)	
	Police	4 (33.3)	
	Both childhood trauma and adult trauma	1 (8.3)	
	Asylum seeker	1 (8.3)	
Exposed to childhood abuse		6 (50.0)	
No previous trauma treatment		7 (58.3)	
Mini International Neuropsychiatric Interview	Major depressive disorder	7 (58.3)	
(*N* = 10)	Panic disorder with agoraphobia	3 (25.0)	
	Obsessive compulsive disorder	2 (16.7)	
	Alcohol dependency	2 (16.7)	
			*M* (*SD*)
Age			44.20 (11.10)
Years after index trauma			18.00 (13.74)
Months of previous treatment			20.83 (27.95)
Baseline CAPS total score^b^			40.75 (7.66)
Baseline PCL-5 total score			47.00 (9.96)
Baseline BSI total score			1.77 (0.65)

^a^Low: completed elementary school or lower vocational education.

Middle: completed high school or middle level vocational education.

High: completed high level vocational education, pre-university, college or university degree.

^b^One patient fulfilled the criteria of the dissociative subtype of PTSD.

**Table 2.** PCL-5 scores of total group (*N* = 12) and in categories of recovered, improved and unchanged.

	T1 Mean (*SD*)	T2 Mean (*SD*)	T3 Mean (*SD*)
PCL-5 total	52.67 (14.34)	42.67 (17.20)	36.25 (20.95)
Recovered (*n* = 2)	49.50 (0.71)	37.00 (25.46)	20.00 (9.90)
Improved (*n* = 7)	50.57 (17.75)	40.29 (17.24)	31.86 (20.65)
Unchanged (*n* = 3)	59.66 (9.29)	52.00 (15.10)	57.33 (9.29)

T1 = day 1; T2 = day 5; T3 = day 21; PCL-5 total score: recovered = no probable diagnosis of PTSD and RC ≤ −1.96; improved = probable diagnosis of PTSD and RC ≤ −1.96; unchanged = probable PTSD diagnosis and RC > −1.96 or <+1.96.

**Figure 1.**
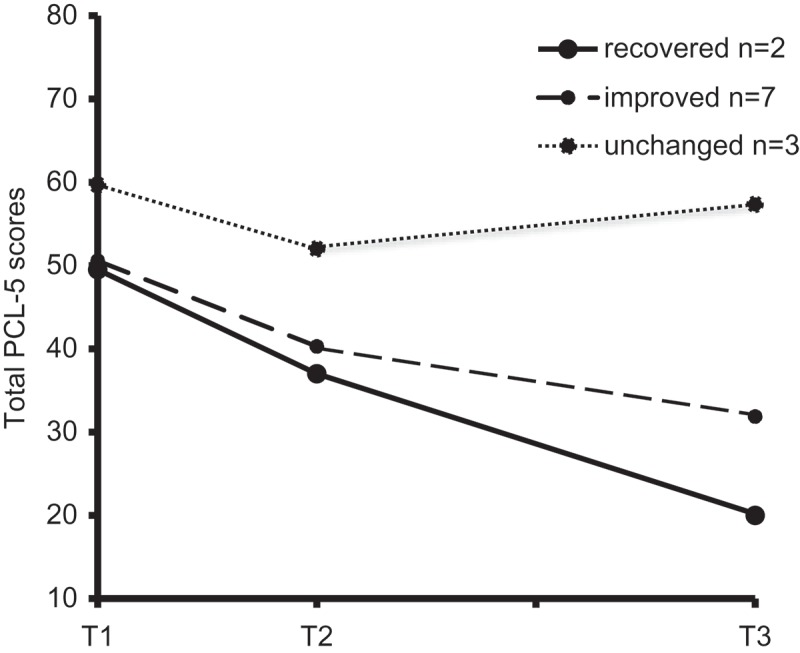
Mean total PCL-5 scores on T1, T2 and T3 in groups of recovered (patient number: 4,7), improved (patient number: 2,11,12,6,1,9,10) and unchanged (patient number: 8,3,5) patients.

Figure 1 shows the mean total PCL-5 scores at each measurement for three participant categories (recovered, improved and unchanged). Mean PCL-5 scores per category are also reported in Table 2. From T1 to T2, the effect was medium-sized (Cohen’s *d* = 0.63). Table 3 shows that nine (81.8%) of the 11 patients who completed treatment reported recovery or improvement between T1 and T3 according to the PCL-5, as indicated by reliable changes. The remaining two patients (18.2%) reported no change with regard to PTSD symptoms between T1 and T3. The 12 patients had a mean RC of −3.13 (*SD* = 2.21) on the PCL-5 between T1 and T3. This effect corresponds with a large effect size (Cohen’s *d* = 0.91). Of the nine patients who showed a reliable change after treatment, two patients showed changes corresponding with a loss of their probable PTSD diagnosis and thus fulfilled the criteria for clinically significant change.

**Table 3.** Treatment outcome and reliable change indexes on PCL-5 scores per patient from T1 to T2 and from T1 to T3.

Patient number	PCL total T1–T2	RCI T1–T2	PCL total T1–T3	RCI T1–T3
4	1	−5.72	1	−6.86
7	3	0.95	1	−4.38
2	3	0.38	2	−4.95
11	3	0.57	2	−2.86
12	1	−7.24	2	−6.48
6	3	−0.76	2	−3.24
1	3	−1.52	2	−2.86
9	3	−1.91	2	−2.48
10	2	−3.24	2	−2.10
8	3	−1.91	3	−0.76
3	1	−2.48	3	−0.38
5	3	-	3	−0.19

T1–T2 = difference between day 5 and day 1; T1–T3 = difference between day 21 and day 1; PCL-5 total score: 1 = recovered (no probable diagnosis of PTSD and RC ≤ −1.96); 2 = improved (probable diagnosis of PTSD and RC ≤ −1.96); 3 = unchanged (probable PTSD diagnosis and RC > −1.96 or < +1.96).

Three of the 12 patients fulfilled the criteria for treatment resistance and had received between 16 and 46 sessions of trauma-focused psychotherapy before enrolment in the intensive programme (BEPP and/or EMDR). Two of these patients responded to the treatment programme with symptom reduction consistent with reliable changes, and one of them prematurely dropped out of the programme. Two other patients had started trauma-focused treatment in an outpatient setting and prematurely stopped this previous treatment because of severe suicidal intent and increase in psychotic symptoms or severe dissociative reactions. Both patients responded to the programme, showing PTSD symptom reduction corresponding with reliable changes.

### 2.1. Feasibility

Tolerability of the sessions is shown in Table 4. The average tolerability score over nine sessions was 4.93 (*SD* = 1.10; range 0–10). None of the patients dropped out because of non-tolerability. The patient who dropped out indicated that he wanted to focus on problems in his relationship. No serious adverse events occurred in the patients who participated in the programme. Three patients reported a mild temporary increase in PTSD symptoms not corresponding with reliable change at T2, and PTSD symptom improvement consistent with reliable changes at T3. One patient reported symptom improvement consistent with reliable change at T2, but not at T3.

**Table 4.** Tolerability of treatment on visual analogue scale (0–10).

	Day 1 Mean (*SD*)	Day 2 Mean (*SD*)	Day 3 Mean (*SD*)	Day 4 Mean (*SD*)	Day 5 Mean (*SD*)
Session 1		4.64 (3.14)	4.58 (3.19)	4.79 (3.94)	5.16 (3.32)
Session 2	4.99 (3.09)	5.63 (3.10)	5.62 (3.76)	3.83 (3.16)	2.66 (2.30)

## 3. Discussion

This pilot study aimed to investigate the feasibility and preliminary effectiveness of a five-day intensive treatment consisting of EMDR, yoga and supportive components. The effects at the last treatment day were modest in terms of PTSD symptom reduction and indicative of a medium effect in terms of effect size. At follow-up, two weeks after treatment, treatment effects were large in terms of effect size and nine of 11 patients reported improvement with regard to self-reported PTSD symptoms corresponding with reliable changes. Two patients no longer met criteria for a probable PTSD diagnosis according to the PCL-5. The other seven patients reported a significant improvement in self-reported PTSD symptoms but still met criteria for a probable PTSD diagnosis. Two patients experienced no change in self-reported PTSD symptoms and one patient dropped out after the first day due to a change in personal treatment goals. None of the patients dropped out due to intolerability and no serious harms or adverse events occurred. Regarding feasibility, the inpatient treatment with EMDR and yoga proved to be acceptable for the large majority of patients who completed the programme and drew benefit from it.

Regarding preliminary efficacy, the large effect size of this uncontrolled study (Cohen’s *d* = 0.91) is encouraging. This effect should be placed in perspective, however, compared to previous meta-analyses of regular weekly treatments and other intensive treatment formats (e.g. Bradley et al., 2005; Ehlers et al., 2014; Hendriks et al., 2010). Bradley et al. found that regular, weekly, trauma-focused treatment resulted in large effect sizes of 1.43. Ehlers et al. reported a very large effect size of 2.45 in their intensive treatment (within group, self-report measurement). The difference in effect size between our study and the study by Ehlers and colleagues may be explained by differences in the format of the intensive treatment programme and characteristics of the included patient populations. Their treatment programme consisted of 18 hours of trauma-focused cognitive therapy over 5–7 days, whereas in our study patients received 13.5–15 hours of EMDR and 4–5 hours of yoga, a total of 17.5–20 hours over five days. This may indicate that a programme with more trauma focused therapy and slightly more time between sessions would be more effective. In the study by Ehlers et al., 36 out of 40 patients experienced their index trauma less than four years ago. In our study, the mean was 18 years (*SD* = 13.74) since index trauma. Time since trauma may reflect other factors related to chronicity that affect treatment outcome (Murray et al., 2017), and may therefore have led to somewhat limited treatment outcome in our pilot study. Another difference in patient population was that half of our sample consisted of military veterans, a population known to benefit less from regular trauma-focused treatments (Steenkamp et al., 2015).

Like in the pilot studies by Hendriks et al. (2010) with TF-CBT and Bongaerts et al. (2017) with EMDR, we found indications that patients with more complex types of trauma and comorbid disorders benefitted from an intensive treatment programme. Although the reason to seek treatment in our pilot study was primarily occupational trauma (10 participants were veterans or police officers), six out of 12 participants also reported traumatic events or neglect during childhood. The programme of Hendriks et al. consisted of five days with six hours of exposure treatment and resulted in large effect sizes (Cohen’s *d* ranging from 1.5 just after treatment to 2.3 at three-month follow-up) in patients who had suffered childhood (sexual) trauma, had high levels of comorbidity and psychosocial stressors and had not previously benefitted from other trauma-focused treatments. The programme by Bongaerts et al. consisted of two times four consecutive days (with a break of three days between) of three hours of EMDR, so a total of 24 hours of trauma therapy; they also found large effects (Cohen’s *d* was 1.7 after treatment, 2.1 at three-month follow-up). These pilot studies thus imply that an even more intensive treatment programme or a two-week programme may be suitable for PTSD patients with a more complex clinical picture.

This pilot study has some important limitations. The first limitation is the lack of a control group, which would have been helpful to assess the effects of time and of nonspecific factors on PTSD symptoms. Also, the sample was small so this warrants caution in generalization of the results. Furthermore, although we assessed both PTSD diagnosis with the latest DSM-5 instrument, the CAPS-5 and also assessed self-reported PTSD symptom severity with the PCL-5 at three time points, the lack of a long-term assessment inhibits conclusions about the stability of the treatment effects. Even though the EMDR sessions were carried out according to the manual by well-trained therapists and tolerability was monitored over sessions, it would be recommended to monitor treatment integrity of each EMDR session. For further research, we also recommend the use of clinician-rated outcome measures for PTSD and comorbidity and optimizing measurement timing. Finally, treatment was offered in a clinical setting, with short individual supportive talks, the encouragement to perform mild exercise and trauma-informed yoga sessions. These additional interventions may have contributed to the treatment effect we found. Especially trauma-sensitive yoga has shown promising results as stand-alone intervention (Van der Kolk et al., 2014) and various forms of physical activity, including yoga, show potential to contribute to effective multidisciplinary treatments for PTSD (Rosenbaum et al., 2015). We cannot completely rule out the possibility that trauma-informed yoga may have lessened the effects of EMDR either, because the effect of the yoga interventions cannot be separated from the effect of the EMDR components in this pilot study. Therefore, no conclusions can be drawn about the separate contribution of various elements.

An important clinical implication is that offering EMDR treatment in this intensive programme seems to be a feasible and fast way to significantly reduce symptoms of PTSD. Patients who fulfilled our definition of treatment resistance, and who had received previous ineffective trauma-focused treatment, drew significant benefit from this intensive treatment. Another clinical implication of this pilot study is that the treatment format can be further developed and optimized. Other intensive treatment programmes indicate that patients may benefit more from a slightly longer (Bongaerts et al., 2017; Ehlers et al., 2014) or even more intensive (Hendriks et al., 2010) treatment format, and including TF-CBT techniques such as exposure in vivo may improve the results. Differences exist between treatment settings (outpatient or inpatient) and no definitive conclusion can be drawn whether the treatment setting matters in the obtained results. Furthermore, for healthcare providers and policy makers it would be interesting to know whether this intensive form is cost- and time-effective compared to weekly sessions.

## 4. Conclusion

In conclusion, this is the first study to investigate the feasibility and preliminary effectiveness of an intensive five-day inpatient treatment with two EMDR sessions and one trauma informed yoga session per day for patients with PTSD. In terms of our objectives, we see it as promising that nine out of 12 patients reported improvement corresponding with reliable changes with regard to PTSD and that none of the patients dropped out due to intolerability. The intensive treatment programme appeared to be effective for patients who were previously unable to benefit from evidence-based treatment, as some of the participants had previously undergone unsuccessful trauma treatment or experienced severe worsening of symptoms in outpatient treatment but responded well to the intensive treatment programme. Although the effect size in our pilot study was large at two weeks post-treatment, we note that the effect size was not as large as in other intensive treatments (e.g. Ehlers et al., 2014) and in regular, weekly trauma-focused treatment (Bradley et al., 2005). Identifying the optimal treatment format and duration is a subject for further study. We would like to recommend more systematic investigation of the effectiveness of intensive treatment for PTSD patients who have not benefitted from regular evidence-based treatments, to compare these intensive formats with regular weekly sessions and to determine the long-term effects. We believe that an intensive, short form of trauma treatment will contribute to a future in which treatment is more tailored to the needs of the individual patient, to ensure the best possible outcome.
